# Artificial Intelligence to Facilitate SEP-1 Measure Compliance and Fluid Management in Sepsis

**DOI:** 10.3390/jcm15093477

**Published:** 2026-05-01

**Authors:** H. Bryant Nguyen, Eduard Krishtopaytis, Enrique Lopez, Neeka Farnoudi, Trinity Van, Viktoriia Kharalampova, Angel Coz Yataco

**Affiliations:** 1 Division of Pulmonary, Critical Care, Hyperbaric, and Sleep Medicine, Loma Linda University, Loma Linda, CA 92354, USA; 2 Department of Medicine, Loma Linda University, Loma Linda, CA 92354, USA; 3 Department of Emergency Medicine, Loma Linda University, Loma Linda, CA 92354, USA; 4 Division of Critical Care, Respiratory Institute, Cleveland Clinic, Cleveland, OH 44195, USA

**Keywords:** artificial intelligence, machine learning, neural network, natural language processing, sepsis, septic shock, fluid resuscitation, antibiotic therapy, bundle performance, SEP-1 measure compliance

## Abstract

Sepsis remains a leading cause of preventable morbidity and mortality worldwide, and adherence to the Centers for Medicare & Medicaid Services Severe Sepsis and Septic Shock Early Management Bundle (SEP-1) remains modest and variable across institutions. Simultaneously, controversy persists regarding fixed-volume fluid resuscitation mandates, particularly given the increasing emphasis on individualized, physiology-guided management. Artificial intelligence (AI) has emerged as a potential strategy to address both operational and clinical gaps in sepsis care. This review examines the current state of SEP-1 implementation, key barriers to compliance, and ongoing debates surrounding early fluid administration. We then discuss contemporary evidence on AI-enabled tools designed to accelerate bundle processes and support personalized fluid management. Early warning systems, natural language processing-augmented models, and telemedicine-integrated platforms have demonstrated improvements in process measures such as time-to-antibiotics and bundle component completion when embedded within defined clinical workflows. Reinforcement learning, causal machine learning, and predictive models offer promise for individualized fluid strategies, although most data remain retrospective and hypothesis-generating. Successful integration will require prospective validation, clinician-in-the-loop oversight, governance frameworks, and continuous monitoring for safety, equity, and model drift. AI should augment—rather than replace—clinical judgment to improve reliability, timeliness, and personalization in sepsis management.

## 1. Introduction

Sepsis remains a leading cause of preventable morbidity and mortality worldwide. Sepsis is a life-threatening organ dysfunction caused by a dysregulated host response to infection, requiring early recognition and coordinated intervention [1,2]. In clinical practice, outcomes depend on a sequence of time-sensitive steps, including recognition, diagnostic confirmation, antimicrobial selection, source control, hemodynamic stabilization, and ongoing reassessment. Failures at any stage can amplify organ injury and increase mortality.

At the turn of the century, the Surviving Sepsis Campaign (SSC) sought to improve survival through standardized pathways emphasizing prompt antibiotics, resuscitation, and hemodynamic support [3]. Early guidelines were strongly influenced by early goal-directed therapy which aimed to formalize time-sensitive resuscitation targets [4,5]. Subsequent multicenter trials did not show superiority of strict hemodynamic targets over contemporary usual care. While these results were in less severe patient populations, SSC updates emphasized clinician judgment, dynamic assessment, and avoidance of downstream harms rather than protocolized care [2]. In the United States, the Centers for Medicare & Medicaid Services Severe Sepsis and Septic Shock Early Management Bundle (SEP-1) accelerated the adoption of guidelines. However, it also raised concerns about measurement reliability, antibiotic stewardship, and the risks of rigid, all-or-nothing protocols in a heterogeneous sepsis population.

Beyond the clinical complexity, sepsis quality improvement has been shaped by bundled processes of care. Early bundle initiatives demonstrated that coordinated, time-dependent actions were associated with improved outcomes, establishing the focus on timeliness and reliability in sepsis [3,6]. However, converting care pathways into performance measures can amplify documentation burden and incentivize checklist behavior when the underlying evidence for specific elements is uncertain or when patient heterogeneity is not fully considered [7,8].

Early detection of sepsis remains a critical first step in any treatment strategy. Clinical signs are nonspecific, and commonly used screening tools (e.g., SIRS, qSOFA, SOFA) have limitations for early identification, especially outside the intensive care unit (ICU) and in patients with chronic organ dysfunction [9]. Artificial intelligence (AI) has emerged as a potential solution. It leverages high-dimensional electronic health record (EHR) data, longitudinal physiologic trends, and unstructured documentation. These models aim to detect deterioration earlier, stratify severity, and provide patient-specific recommendations. This may shorten time to appropriate therapy. AI is being explored not only for early sepsis prediction but also to support implementation—routing the right information to the right team at the right time, reducing cognitive load, and promoting consistent, evidence-based care while preserving clinician oversight [10,11].

In this article, we discuss the current state of SEP-1 implementation, specifically as it relates to fluid resuscitation. We then explore how AI-enabled tools may (1) reduce gaps in sepsis guidelines adherence and SEP-1 compliance by delivering actionable, workflow-concordant decision support, and (2) guide individualized fluid management given ongoing equipoise and practice variation in septic shock. Finally, strategies and future directions for AI integration in sepsis management are proposed. We refer the reader to other resources on the use of AI in prediction, prognostication, and overall management of sepsis [12,13,14]. Our review will focus on the SEP-1 measure and early fluid resuscitation.

## 2. Review Methodology

A literature review was conducted to synthesize current evidence on AI applications in sepsis management. Relevant studies were identified through targeted searches of PubMed, Google Scholar, and Scopus, prioritizing publications from 2020 to the present. We included studies describing AI-based sepsis prediction, decision support, and hemodynamic optimization in sepsis, with emphasis on SEP-1/sepsis bundle compliance and fluid resuscitation. Priority was given to studies reporting workflow integration, process measures, or patient-centered outcomes. Reference lists of identified articles were also reviewed to identify additional relevant studies. Given the heterogeneity of study methodologies and outcomes, a formal systematic review was not performed.

## 3. Current State of SEP-1 Sepsis Measure Compliance and Implementation Barriers

National SEP-1 performance has remained modest and heterogeneous since implementation. Mean hospital compliance is approximately 49%, with wide interhospital variability [15]. In an emergency department network, mean compliance was approximately 54% with substantial variation across bundle components, particularly intravenous fluid administration, and repeat lactate measurement [16]. However, these aggregate metrics obscure the clinical reality that sepsis presentations and care pathways vary widely across emergency, ward, and ICU settings.

SEP-1 requires a complex chart review and subjective determination of sepsis “time zero,” creating potential for misclassification and inter-hospital noncomparability. In a study by Rhee et al. across three hospitals, abstractors agreed on “time zero” in only 36% of cases, and calculated pass rates differed substantially across reviewers [8]. Such variability limits interpretability, complicates benchmarking, and may blunt quality-improvement feedback.

Structural and organizational factors may also influence performance. A nationwide study linked lower odds of SEP-1 compliance to larger hospital size and teaching status, suggesting that case-mix complexity, competing priorities, and operational load may interact with performance [17]. SEP-1’s administrative burden and prescriptive timing rules have also been criticized. From the clinician perspective, strict requirements may incentivize unnecessary broad-spectrum antibiotics or fixed-volume fluid administration in patients with tenuous cardiopulmonary reserve, shifting attention from physiology-driven resuscitation to documentation-driven completion [7,18].

SEP-1 was designed to measure time-sensitive care, but its complexity creates a substantial abstraction burden. The measure links several required elements: initial lactate measurement, blood cultures before antibiotics, timely broad-spectrum antibiotics, a defined crystalloid bolus for hypotension or hyperlactatemia, vasopressors for persistent shock, and repeat lactate measurement. Each element is tied to a strict time window [7,19]. Failure of any single component can render the entire case noncompliant. As a result, hospitals may focus not only on clinical care redesign but also on documentation and coding strategies. This dynamic can shift the emphasis away from bedside decision-making. Clinicians may therefore experience SEP-1 as a compliance exercise rather than a patient-centered framework [7].

In this context, interventions that improve performance on discrete components, such as early antibiotic administration or lactate reassessment, may be more actionable than pursuing all-or-none compliance. However, individual elements can vary in feasibility obscuring true performance [15,16]. Many health systems therefore pursue targeted workflow redesign—standardized triage, early order sets, and escalation pathways—while acknowledging that strict SEP-1 abstraction may not fully capture clinical judgment in complex cases, particularly when fluid dosing or antibiotic timing decisions need to be individualized [8,19].

Given the challenges, associations of SEP-1 compliance with outcomes are debated. In a large propensity score matched cohort of Medicare beneficiaries, SEP-1 compliant care was associated with lower 30-day mortality compared with noncompliant care, supporting the plausibility that timely, coordinated processes matter [19]. Conversely, other work emphasizes that SEP-1 failure may reflect clinical complexity (e.g., hospital-onset sepsis, atypical presentations) rather than poor care. Additionally, some mandated elements lack strong outcomes evidence [7,8]. These debates motivate decision-support strategies that improve timeliness and reliability while preserving flexibility for patient-centered, physiology-guided care.

## 4. Ongoing Fluid Resuscitation Controversies in Septic Shock

The long-standing recommendation for an initial fixed-volume 30 mL/kg crystalloid bolus in septic shock has been increasingly challenged. Critics argue that septic shock is not uniformly a hypovolemic state and that indiscriminate fluid loading may exacerbate vasoplegia, myocardial dysfunction, endothelial injury, and tissue edema, thereby impairing oxygen delivery despite increased intravascular volume [20]. Thus, the central risk is not “too much fluid” per se, but physiologically mismatched therapy in distributive shock patients with impaired vasomotor tone without preload deficiency.

Concerns about harm have been observed by studies in resource-limited settings. In a clinical trial completed in Africa, aggressive fluid strategies for septic patients were associated with worsening respiratory status and mortality, underscoring that one-size-fits-all approach should not be standard practice [21]. Although these settings differ from high-resource ICUs, the findings illustrate that physiologic context and monitoring capacity can modulate the risk–benefit profile of early fluid resuscitation.

In contemporary high-resource settings, recent randomized trials in emergency department and intensive care unit populations have not shown a mortality benefit for restrictive versus liberal fluid strategies after initial resuscitation [22,23]. In patients with septic shock in the ICU, Meyhoff et al. found that a restrictive strategy did not reduce mortality compared with usual care but did reduce cumulative fluid exposure [22]. In the emergency department (ED) patients with early sepsis-induced hypotension, Shapiro et al. showed that earlier vasopressor use with lower fluid volumes resulted in similar mortality and clinical outcomes as a liberal-fluid approach, supporting a physiology-guided strategy [23]. The trial was stopped early for futility after enrollment of approximately two-thirds of the target population. Although outcomes were similar, earlier vasopressor use was associated with increased intensive care resource utilization.

Systematic reviews and meta-analyses additionally found no evidence that fluid restriction or fluid de-resuscitation is superior to higher fluid volumes for mortality in septic shock, underscoring uncertainty about optimal cumulative fluid balance strategies [24,25,26]. Guideline and expert perspectives have therefore shifted toward individualized resuscitation. Contemporary recommendations emphasize early hemodynamic assessment, and ongoing reassessment to avoid fluid overload after stabilization [27]. Practical bedside frameworks highlight dynamic assessment of fluid responsiveness and tolerance; e.g., passive leg raise with stroke volume response, echocardiographic surrogates of preload responsiveness, and careful monitoring of venous congestion and oxygenation [28]. An example of such specific approach is the ANDROMEDA-SHOCK-2 trial where clinicians administered crystalloid boluses only to “fluid responders” [29]. For patients with a distributive shock profile, reflected by pulse pressure ≥ 40 mm Hg and diastolic blood pressure < 50 mmHg, vasopressors are initiated early to mobilize the stressed volume before further fluids. Study results show shorter duration of organ support in the personalized resuscitation group despite lower fluid volumes.

The controversies in fluid management are directly relevant to performance measures. While previous studies examining restricted versus liberal fluid strategies had patients enrolled after an approximately 30 mL/kg of crystalloid fluid administration, fixed-volume mandates embedded in SEP-1 may conflict with individualized, physiology-driven care, particularly for patients at elevated risk of fluid-related harm [22,23]. This tension strengthens the rationale for decision support, potentially AI-enabled, which identifies patients most likely to benefit from fluids, recommends response- and tolerance-informed fluid amount, and facilitates documentation when clinically appropriate deviations from prescriptive elements occur.

## 5. AI Potential for Clinical Decision Support in Critical Care

AI research on critical care has expanded rapidly, with literature searches demonstrating accelerated growth in publications and applications over the last decade [11,30]. Across ICU applications, predictive analytics dominate, particularly for the early detection of deterioration and outcome prognostication [31]. In parallel, methods for sequential decision-making (including reinforcement learning [RL]) have been applied to treatment policy optimization, motivated by the ICU’s high-frequency data streams and the need for time-dependent decisions.

AI-enabled clinical decision support in the ICU typically integrates time-series physiologic data, laboratory values, medication exposures, and structured EHR variables to support risk stratification and actionable alerts. Moazemi et al. performed a systematic review and identified twenty-one studies for qualitative analysis, focusing on adult cardiovascular ICU populations and AI/ML models applied to clinical time series and EHR data, most commonly using methods such as gradient boosting, recurrent neural networks, and reinforcement learning. Results showed generally strong predictive performance (AUROC ~0.79–0.96), but highlighted major limitations including lack of external validation (≈75% of studies), poor generalizability, and limited interpretability [32].

Despite rapid methodological innovation, AI in the ICU remains vulnerable to translational failures. Models may perform well during development but degrade over time with changes in documentation, clinical practice, patient mix, or monitoring technology (“model drift”). In addition, data labels are sensitive to evolving sepsis definitions and differences in patient populations (“data shift”) [33,34]. Data shift and demographic bias can cause sepsis AI models to perform worse in hospitals or patient groups that differ from the populations on which they were trained, producing unequal sensitivity, calibration, and false-alert rates across subgroups. This can worsen disparities by delaying recognition and treatment in some patients while increasing unnecessary alerts in others, underscoring the need for external validation, subgroup-level evaluation, and ongoing model recalibration.

Many automated systems also contribute to alert fatigue. High rates of false positives erode trust and reduce clinical responsiveness. To mitigate these risks, several strategies can be used. These include careful threshold calibration, tiered notifications, and context-aware suppression (for example, during ongoing resuscitation). Sendak et al. performed a single-center implementation study integrating a deep learning-based sepsis detection platform into the emergency department of an academic health system. They used EHR time-series data from ~39,918 adult ED encounters to develop and deploy a multitask Gaussian processes (MGPs)–recurrent neural network (RNN) model within a real-time clinical workflow. The system was successfully implemented with high clinician engagement and continuous risk prediction, demonstrating feasibility of real-world integration but requiring substantial infrastructure and organizational coordination [10].

Systematic reviews show that most ICU AI studies remain retrospective, with limited external validation and scarce evidence on patient-centered outcomes after real-world deployment [35]. Gallifant et al. performed a systematic review evaluating the design, reporting quality, and risk of bias of AI applications in mechanical ventilation by analyzing ninety-five studies across MEDLINE, Embase, and PubMed. Most studies were single-center retrospective analyses (88%) with high risk of bias (89%) and limited data/code availability. Reporting and reproducibility challenges were also observed, including incomplete transparency in model development, inadequate handling of missing data, and insufficient attention to calibration and post-deployment performance monitoring [36]. These limitations highlight that model performance is necessary but insufficient. Robust deployment requires workflow design, governance, and continuous monitoring to ensure safety and sustained benefit.

Table 1 further outlines the major domains of challenge affecting the safe and effective use of AI in critical care, spanning evidence quality, data generalizability, model transparency, clinical integration, ethical considerations, and long-term sustainability.

**Figure 1:**
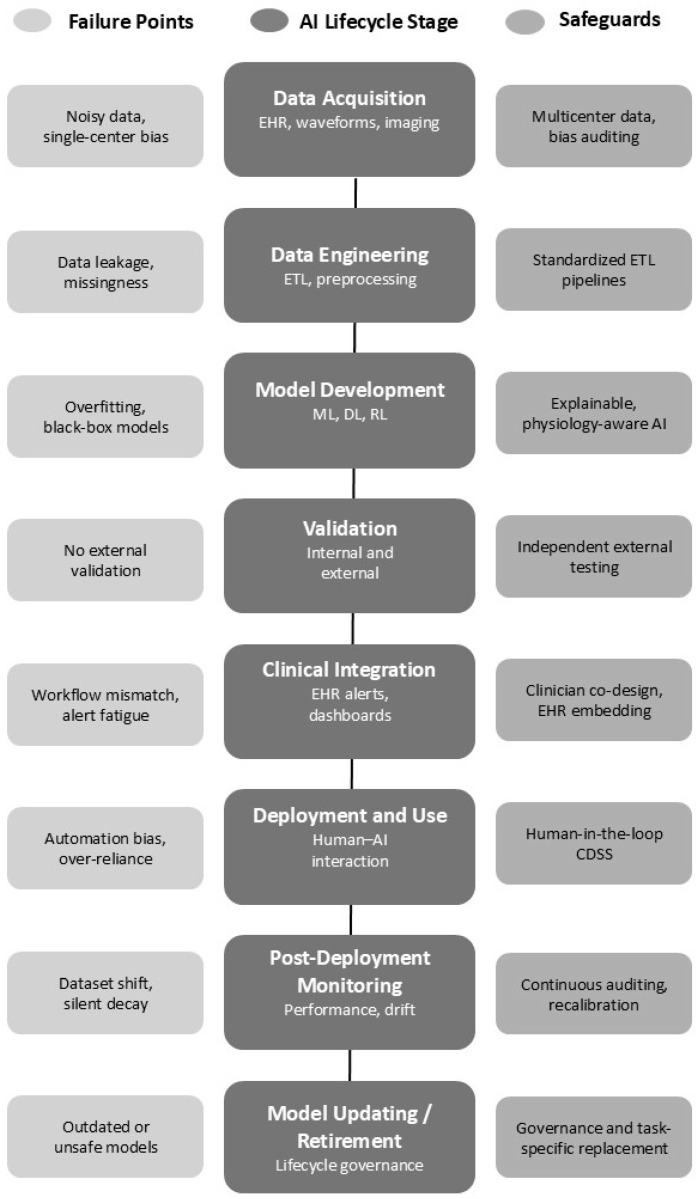
Artificial intelligence lifecycle in critical care medicine highlighting key failure points and safeguards [35,37,38,39,40,41,43,44].

Figure 1 illustrates the end-to-end AI lifecycle in critical care, from data acquisition through deployment and monitoring, emphasizing where failures most commonly occur, such as data bias, model drift, lack of interpretability, and poor clinical integration. These critical failures can be attenuated by safeguards including governance structures, human-in-the-loop oversight, external validation, and continuous performance monitoring.

## 6. AI for Improving Sepsis Bundle Performance and SEP-1 Compliance

AI models intended to improve sepsis bundle performance begin with earlier and more reliable recognition of sepsis. Automated early warning systems using routinely collected EHR variables can screen broad inpatient populations continuously, potentially reducing missed cases and delays. For example, Cooper et al. developed and implemented an automated sepsis screening tool in a 255-bed community hospital using logistic regression based on six clinical variables, derived from a retrospective cohort of 10,792 hospitalizations (including 339 sepsis cases) and validated on a separate cohort, followed by prospective and real-time implementation. The model demonstrated good discrimination (AUROC ~0.85) and enabled automated, real-time screening and early detection of 100% of inpatients, contributing to reduced sepsis mortality [45].

More complex systems have incorporated gradient boosting, neural networks, and ensemble methods to identify sepsis risk hours before clinical recognition, providing a larger window for interventions [46]. Goh et al. developed and validated an AI-based sepsis early risk assessment algorithm (SERA) in a hospital setting in Singapore by integrating structured EMR data with unstructured clinical notes using natural language processing, trained and tested on 5317 patients (114,602 clinical notes) with defined sepsis cohorts based on ICU admission and ICD-10 codes. The model achieved high predictive performance (AUROC up to 0.94) for both diagnosis and early prediction at 12 h before onset, outperforming physicians, and traditional scoring systems, increasing early detection by 32% while reducing false positives up to 17% [47].

However, predictive accuracy alone rarely ensures clinical impact. Models must be embedded in the patient care workflow that is timely, role-based, and interpretable so that the alerted clinicians can respond quickly. An early warning system, *Sepsis Watch*, can pair model output with rapid response team nurse review and escalation pathways, reframing AI output as a trigger for needed responses rather than an interruptive alert, which at times may be ignored. This approach was associated with improvements in time-to-antibiotics and other process measures [10,48].

Telemedicine may similarly extend surveillance and clinician responsiveness. Gaieski et al. performed a prospective observational study to evaluate the impact of implementing an end-to-end *Telesepsis* solution—including EMR-linked automated monitoring, nurse navigators, and teleconsultation—in five emergency departments within an academic health system [49]. They enrolled adult patients screened for sepsis based on clinical criteria during a surveillance and intervention period. Among over 56,000 ED encounters (including 1233 confirmed sepsis cases), the intervention significantly improved SEP-1 bundle compliance (from 68.4% to 78.3%, *p* = 0.002) and enhanced real-time identification and management of sepsis patients compared to the baseline surveillance period.

Several studies have further evaluated different modeling paradigms for early sepsis recognition. A deep learning approach using recurrent neural networks predicted sepsis onset hours in advance within ICU cohorts, demonstrating that model-driven alerts could theoretically increase lead time for intervention [50]. More recent work has emphasized model calibration and bedside usability. For example, approaches that combine gradient boosting with feature attribution or rule extraction aim to increase interpretability so that clinicians can understand what is driving a high-risk alert [51,52].

Studies of sepsis alerts frequently report improvements in process measures such as time-to-antibiotics or completion of key bundle elements, particularly when these alerts are coupled to structured triage, escalation, and rapid clinician response. Conversely, alerts that are poorly integrated into workflow can increase interruptions, contribute to unnecessary antibiotics, and shift attention away from other competing tasks. Implementation therefore must include guardrails, accountability of alert response, and balancing outcomes [10,53].

Bundle compliance is an all-or-none construct, so missed documentation or a single delayed element (e.g., repeat lactate) can imply failure despite appropriate care. Wells et al. performed a scoping review evaluating the extent and depth of SEP-1 education in acute care settings [54]. They analyzed twenty studies focusing on clinician training interventions and their impact on compliance and outcomes. While most studies emphasized sepsis recognition and bundle elements, they lacked “in-depth” education on documentation of clinical judgment and real-time decision support (e.g., checklist-driven workflows), with only 5% addressing these elements. Their study highlighted that insufficient documentation support and real-time checklist integration contribute to stagnant compliance rates despite some reported improvements. Across studies, impacts on outcomes are mixed and remain difficult to generalize. A systematic review of machine learning sepsis prediction implementations found that only a minority demonstrated statistically significant reductions in mortality, and many reported primarily algorithm performance and process improvements rather than patient-centered outcomes [46].

A potential limitation in clinical prediction modeling is temporal data leakage, where model inputs inadvertently include information not available at the time of decision-making. For example, using the time a *lactate* laboratory result becomes available rather than when it was ordered earlier may artificially underestimate model performance. Studies should align variable timestamps with real-time clinical workflows to avoid this bias. Nevertheless, the consistent result that automated surveillance can shorten time-to-treatment supports the assertion that AI can improve bundle completion, particularly when coupled with implementation strategies that translate patient risk into timely action.

Table 2 further illustrates representative studies evaluating AI-enabled systems designed to improve sepsis recognition and sepsis bundle performance. The included interventions span EHR-embedded clinical decision support tools, telemonitoring/telesepsis workflows, and machine learning/deep learning models (including NLP-augmented approaches), with reported benefits most consistently observed in process metrics such as tool utilization, alert-to-action engagement, SEP-1 compliance, and time-to-antibiotic administration. While many studies report improvements in process measures, these results do not necessarily translate into patient-centered outcomes. A few studies have shown that AI sepsis prediction models were associated with decreased patient mortality; however, they were observational cohorts or before-and-after designs [55,56,57]. Prospective randomized evidence is necessary to demonstrate that improvement in process measures results in improvement in survival and other patient-centered outcomes.

## 7. AI-Assisted Fluid Resuscitation for Septic Shock

AI-assisted hemodynamic decision support for sepsis spans three broad approaches: (1) forecasting fluid requirements, (2) estimating fluid responsiveness using multimodal physiologic data, and (3) learning sequential policies for fluid decision-making using reinforcement learning (RL) or causal/counterfactual methods. Table 3 summarizes studies applying AI to fluid and hemodynamic management in sepsis, spanning early predictive models, supervised machine learning tools for fluid responsiveness/urine output response, and prescriptive approaches using reinforcement learning, counterfactual reasoning, causal machine learning, and automated control frameworks.

Early work demonstrated the feasibility of probabilistic forecasting. Celi et al. performed a proof-of-concept study aimed to evaluate the feasibility of using AI to predict individualized fluid requirements in ICU patients by analyzing data from the MIMIC II database at a single academic center, including 3014 patients on vasopressors [61]. Regression and Bayesian network modeling of demographic and high-resolution physiologic variables from the first 24 h were used to predict fluid needs on ICU day two. The Bayesian network model achieved a predictive accuracy of 77.8%, demonstrating that AI-driven analysis of early ICU data can estimate subsequent fluid requirements, supporting its potential role in personalized, data-driven critical care decision-making.

Predicting fluid responsiveness is another active area of research. In a prospective observational study, Bataille et al. aimed to evaluate whether machine learning models could improve prediction of fluid responsiveness in patients with severe sepsis or septic shock in a single-center ICU [69]. They enrolled one hundred patients over two years using transthoracic echocardiography data (baseline, passive leg raise, and post-fluid challenge) to train and test multiple algorithms. Machine learning models—particularly partial least-squares and neural networks—demonstrated strong predictive performance (test AUROC ~0.83–0.85), comparable to passive leg raising. Their results identified key echocardiographic predictors and supported AI-based approaches as viable tools for bedside fluid responsiveness assessment.

Other predictive models have been explored to forecast patient-specific responses to fluids, leveraging time-series vital signs, laboratory trends, and evolving organ dysfunction to anticipate treatment trajectory rather than relying on a single snapshot [14,42]. Combined bedside ultrasound features, laboratory, and clinician documentation have also been examined to predict tolerance of additional fluids and risk of fluid overload [14,42]. Models incorporating waveform-derived features have shown strong performance in predicting volume responsiveness, potentially enabling continuous monitoring rather than intermittent assessments [70]. Additionally, phenotype-based methods to identify patient subgroups with different fluid tolerance and responsiveness could assist clinicians in choosing between fluids and earlier vasopressor support [72].

More recent research has shifted toward sequential decision-making, in which the patient’s condition evolves over time and clinical actions influence future physiologic changes. Komorowski et al. developed and validated a reinforcement learning-based clinical decision support model (“AI Clinician”) to optimize fluid and vasopressor therapy in sepsis patients [62]. Their study used retrospective data from two large ICU databases (MIMIC-III and eICU-R1) including 96,156 adult sepsis patients, with time-series modeling of forty-eight clinical variables and Markov decision processes to learn optimal treatment policies. The AI-derived treatment policy demonstrated higher expected survival benefit than clinician decisions. Patients whose treatments aligned with AI recommendations had the lowest mortality, with lower fluid and earlier/higher vasopressor use compared to usual care.

A complementary line of work focuses on individualized fluid dosing at the bedside. A study by Gupta et al. aimed to develop a human-in-the-loop artificial intelligence model to optimize patient-specific intravenous fluid resuscitation in sepsis [63]. Their study included a retrospective cohort of 1122 ICU patients with sepsis extracted from the MIMIC-III database. Inverse classification with machine learning (logistic regression and neural networks) embedded in a constrained optimization framework was applied. The model demonstrated that AI-guided, physician-informed fluid recommendations could reduce mortality risk, with results showing an approximate 22% relative reduction in predicted mortality compared to baseline treatment strategies, highlighting the benefit of combining clinician input with AI-driven optimization. Such approaches align with contemporary resuscitation controversies: rather than fixed fluid bolus, the goal is to recommend patient-specific doses based on evolving physiology and responses.

Another approach draws from control theory and automated titration. An automated fluid resuscitation framework using a variational autoencoder (VAE)-based nonlinear state-space model combined with model predictive control (MPC) was developed by Estiri et al. to optimize hemodynamic responses [73]. They used data from an animal hemorrhage model involving sheep with recorded MAP responses to fluid infusion and bleeding over time. The VAE-based MPC system accurately predicted MAP dynamics, achieving more stable and precise control of blood pressure and fluid dosing. These systems are attractive because they can encode explicit constraints (e.g., limits on vasopressor dosing, cumulative fluid amount, and safety thresholds for oxygenation and lactate levels) and optimize multiple objectives in the presence of uncertainties.

Across approaches, the dominant limitation is translational readiness. Sepsis AI tools are sensitive to data labeling choices, confounded by treatment indications, and changes in practice over time that can degrade accuracy and transportability [71,74]. RL and counterfactual evaluations rely on assumptions embedded in off-policy estimators and may not generalize across settings or evolving practice patterns [62,71]. RL models rely on retrospective clinician-generated data and assume no unmeasured confounding, which is rarely true. Unobserved human factors not recorded in the EHR such as clinician gestalt may bias recommendations. For bedside adoption, models must also provide rationale, integrate with clinician workflows, and be evaluated prospectively with safety-focused endpoints before being used to guide hemodynamic resuscitation.

A crucial methodological challenge is causal inference. Retrospective datasets used to develop AI models capture what clinicians had chosen to do, rather than what would have occurred under alternative treatment actions. Reinforcement learning and counterfactual methods estimate the value of treatment sequences but are at risk of unmeasured treatment variability and clinician selection bias. Consequently, most algorithm outputs should be interpreted as hypothesis-generating rather than prescriptive, reinforcing the need for clinician-in-the-loop decision support rather than autonomous control. This caution is further underscored by the fact that, while “AI clinicians” have shown promise, the absence of prospective randomized controlled trials remains a major barrier to clinical adoption. Accordingly, prospective evaluation should prioritize safety, calibration to local practice, and physiologic plausibility [62,63,75].

**Figure 2:**
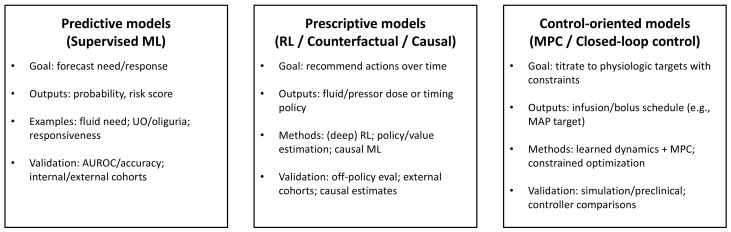
Taxonomy of AI approaches for fluid and hemodynamic management in sepsis patients. Predictive models estimate fluid requirement or physiologic response. Prescriptive models recommend time-varying fluid and/or vasopressor strategies. Control-oriented models aim to titrate therapy to physiologic targets [61,62,63,64,65,66,67,68,69,70,73,76].

Figure 2 proposes a taxonomy of AI approaches for fluid and hemodynamic management in sepsis. Predictive models can estimate fluid requirement or physiologic response. Prescriptive models will recommend time-dependent fluid and/or vasopressor strategies. Finally, control-oriented models aim to titrate therapy to optimal physiologic targets.

## 8. Strategies for AI Implementation in Sepsis Management

Implementation barriers for sepsis AI cluster around trust and interpretability, workflow integration, technical readiness and generalizability, governance and safety, leadership alignment, and the resources required for sustainment (Table 4) [10,33,34,46,48,77,78].

Joshi et al. examined implementation approaches and barriers for rule-based and machine learning-based sepsis clinical decision support tools across 15 United States medical centers, using semi-structured interviews and questionnaires from 21 hospital leaders involved in CDS deployment [77]. Their study found that implementation was complex and resource-intensive, with major barriers including alert optimization, workflow integration, and especially clinician acceptance. There were greater distrust, confusion, and interpretability challenges for machine learning models, highlighting the need for improved user education, transparency, and implementation strategies. Addressing acceptance requires transparent reporting, clear definition of intended use, and alignment between model outputs and human judgment.

Workflow integration is equally important, as even accurate models can fail when alerts are misaligned with clinical roles, timing, and decision points or when they exacerbate alert fatigue. Sandhu et al. evaluated the integration of a machine learning-based sepsis early warning system into clinical workflows at a large academic medical center, using semi-structured interviews with fifteen frontline clinicians (seven emergency physicians and eight rapid response team nurses) [48]. The study found that successful implementation depended on clinician trust, workflow integration, and human-mediated communication. AI systems improve vigilance and facilitate sepsis recognition, but key barriers included limited model understanding, information flow challenges, and concerns about accuracy and interpretability.

*Sepsis Watch* illustrates a micro-workflow approach: high-risk alerts were triaged by trained rapid response team nurses who reviewed charts, contacted bedside teams, and escalated to physicians, thus coupling an AI output with a defined clinical response [10,48]. Implementation studies similarly emphasize that pairing alerts with escalation protocols is essential to translate prediction into improved process measures and outcomes. However, technical barriers arise upstream, including data quality issues, limited interoperability, and lack of diversity in training datasets, all of which can degrade calibration and equity when models are generalized across sites [33,34]. External validation should assess both overall performance and performance across patient subgroups. Models trained in tertiary academic centers may perform differently in community hospitals and may introduce bias if not carefully evaluated. Equity-focused audits should examine performance across comorbidities, age, sex, and socioeconomic or racial groups to ensure that implementation does not exacerbate health disparities.

Governance concerns include unclear liability, privacy requirements, and unintended harms such as unnecessary antibiotics or fluids triggered by false positives [78]. Practical mitigation strategies include rigorous external and temporal validation, prospective silent trials before deployment, post-implementation monitoring for model drift and disparities, and safety constraints that prioritize “do no harm.”

Equity and drift are increasingly recognized implementation risks. Models trained on historical data may encode disparities in access to care, documentation, or monitoring intensity, resulting in unequal performance across race, language, or socioeconomic groups [34]. Additionally, model drift occurs when changes in clinical practice or patient populations alter the relationship between model inputs and outcomes, leading to performance degradation. Even when initially well calibrated, models can degrade as sepsis definitions, treatment patterns, or antimicrobial stewardship practices evolve. Van der Vegt performed a systematic review to evaluate real-world deployment of machine learning-based sepsis prediction algorithms across adult hospital settings [46]. They analyzed thirty studies (2015–2022), including implemented models utilizing live or near-live EHR data and assessing performance, implementation factors, and outcomes. Across studies, post-implementation performance was inconsistently reported and often declined compared to pre-deployment metrics, reflecting the impact of data and model drift due to evolving clinical practices and data variability, underscoring the need for continuous monitoring, recalibration, and lifecycle evaluation to maintain model reliability in real-world settings.

Automation bias also represents a critical concern, as clinicians may over-rely on AI recommendations even when they conflict with bedside findings. Accordingly, human-in-the-loop frameworks and targeted clinician training are essential. AI should be framed as a tool that augments, rather than replaces, physician clinical judgment, with clinician training focused on preserving independent reasoning. Recent guidance and human-factors literature emphasize that safe clinician–AI interaction requires human oversight, calibrated trust, awareness of model limitations, and deliberate verification of AI outputs against the patient’s examination, physiology, and clinical trajectory [79,80]. AI can worsen performance when its recommendations are biased or incorrect. In a randomized vignette study, clinicians exposed to a systematically biased model had lower diagnostic accuracy, and explainability features did not fully mitigate this effect [81,82]. Accordingly, clinician training should teach AI as a hypothesis-generating aid rather than an authority, using strategies such as independent first-pass assessment, structured contradiction checks, simulation with intentionally erroneous AI recommendations, and diagnostic time-outs when AI outputs conflict with bedside physiology, thereby reinforcing that the final integrative judgment remains the physician’s responsibility [79,80,83].

**Figure 3:**
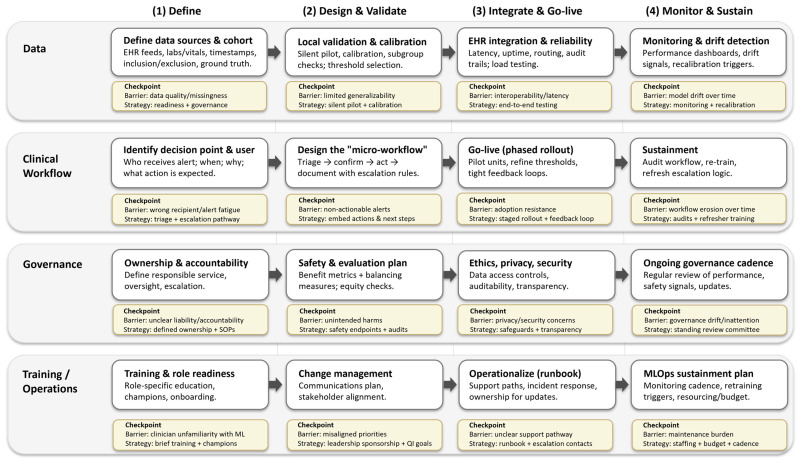
AI sepsis tool implementation pathway with workflow phases (planning, implementation, and sustainment) across socio-technical domains (data, clinical workflow, governance, and training/operations). Checkpoint callouts summarize common barriers and paired mitigation strategies reported in real-world implementations and systematic reviews [10,33,34,46,48,77,78].

Figure 3 provides a potential framework for deploying AI-enabled sepsis decision support that involves data, clinical workflow, governance, and training. Each work stream progresses through four sequential phases from definition, design and validation, integration and go-live, to monitoring and sustainment. Each step must also include mitigation strategy for common implementation barrier. Successful sepsis AI deployment therefore depends as much on workflow, governance, and sustainment infrastructure as on model performance.

## 9. Future Directions of AI in Sepsis Management

Short-term impact of AI in sepsis management will depend on implementation strategies which should aim to improve SEP-1 performance and individualize fluid management. Prospective studies should evaluate both process and patient-centered outcomes. Process endpoints include time to antibiotics, timely blood cultures, lactate measurement and re-measurement, cardiovascular support, and achievement of hemodynamic goals. At the same time, studies must explicitly monitor adverse effects, such as unnecessary antibiotic use, excessive or inadequate fluid administration, increased ICU utilization, and resource strain [10,46]. In parallel, a “measurement-aware” model design is needed. Prediction targets should align with guideline-relevant clinical decisions. Finally, models should reduce documentation burden and improve SEP-1 performance.

For fluid and vasopressor guidance, future work should align with ongoing clinical equipoise by prioritizing clinician-supervised, constraint-aware systems that provide recommended dosing ranges, considering clinical uncertainties and physiologic rationale rather than prescriptive instructions [27]. Phenotyping may identify subgroups with differing fluid tolerance and responsiveness, enabling “right patient, right fluid” strategies that reconcile quality measures with individualized care [72]. Scalable benefit will require not only better algorithms, but also robust governance frameworks, multi-site evaluations, and implementation designs that reliably translate AI outputs into clinical actions.

Future tools should link predictions to clinically interpretable targets: identifying fluid responders, forecasting hypotension, suggesting timing of vasopressor initiation, and supporting safe de-escalation as shock resolves. Multimodal inputs, such as bedside ultrasound, invasive hemodynamic monitoring, and ventilator waveform data, may result in more accurate recommendations than using static EHR variables [9,72]. Prospective studies should include subgroup and diversity analyses, explicit measurement of clinician burden, and pre-specified monitoring for unintended harm, given the heterogeneity of sepsis phenotypes and the risk of varying performance across sites and populations [46,78].

Methodologically, the adoption of AI would benefit from common reporting standards, external validation across diverse health systems, and head-to-head comparisons of AI screening versus clinician judgment using clinically meaningful endpoints rather than AUROC alone [10,33]. While many studies report model discrimination using AUROC, this metric alone does not fully capture clinical utility. Model performance is influenced by disease prevalence, calibration, and clinical context. For example, a model with a high AUROC may still have limited practical value in low-prevalence settings because the positive predictive value remains low. Interpretation of model performance should therefore consider calibration and clinical applicability in addition to discrimination. Effective implementations can evaluate whether AI improves processes and outcomes while simultaneously identifying barriers to adoption, including alert burden, staffing constraints, and unintended treatment effects such as unnecessary antibiotics or excessive fluids. Finally, post-deployment evaluation should be ongoing, including monitoring model drift, recalibrating thresholds, auditing equity, and ensuring that model updates are governed in ways that preserve trust and clinical accountability [34,78].

From a systems perspective, future sepsis AI models should be evaluated as an intervention bundle, including elements such as algorithm performance, workflow design, staffing requirement, escalation protocol, and patient-centered outcomes. Implementation science frameworks can help specify who receives alerts, what actions are expected, and what resources are required to respond, thereby reducing alert fatigue and ensuring accountability [10,77].

## 10. Summary and Conclusions

Sepsis care is time-sensitive yet heterogeneous, creating conflict between guidelines and individualized treatments. National SEP-1 performance remains modest and variably measured. While some studies associate compliance with improved outcomes, measurement variability and limited evidence for certain mandated elements remain central concerns [7,8,15,19]. These gaps require strategies that improve timeliness and reliability without enforcing standardization. Accordingly, AI should be evaluated to improve reliability and augmenting clinical decision-making, not as a substitute for patient-specific resuscitation.

AI-enabled early warning systems and EHR-integrated clinical decision support can improve surveillance, risk stratification, and the timeliness of key sepsis processes required by SEP-1 [45,48,49]. For hemodynamic management, contemporary trials support equipoise between liberal and restrictive early fluid strategies, reinforcing the rationale for adaptive, physiology-guided resuscitation [22,23]. AI-based approaches offer a plausible pathway to individualize fluid resuscitation. However, most available evidence remains retrospective and hypothesis-generating. Although the use of decision-support systems at this stage is appropriate, autonomous, and unsupervised application will require stronger evidence.

Prospective, safety-focused studies, along with careful implementation and evaluation, will be essential to determine whether AI tools can reliably address gaps in sepsis care while preserving clinical judgment and patient-centered decision-making. An evidence-based evaluation of an AI model would extend far beyond reporting accuracy with an AUROC. The process would begin with formal clinical trial registration and clearly pre-specified endpoints. The trial protocol would include a comprehensive data supplement detailing variable definitions, data extraction methods, approaches to missing data, specific AI algorithms used, and safeguards to prevent temporal data leakage. This would be followed by a prospective, randomized controlled trial assessing not only process measures, such as time to antibiotics, but also patient-centered outcomes and important balancing measures related to potential harm. After deployment of the AI model, evaluation would remain ongoing, supported by a publicly available model performance reporting mechanism over time and across patient subgroups to promote transparency and accountability.

## Figures and Tables

**Table 1:** Key issues, evidence, and literature-supported solutions for the challenges with AI applications in critical care.

Domain	Key Issue	Evidence/Description	Proposed Solutions
**Evidence Quality**	Retrospective, biased study designs	>95% of studies retrospective; 80–90% high risk of bias; limited outcome evaluation	Prospective, multicenter pragmatic trials; TRIPOD+AI 2024/CONSORT-AI Extension 2020 adherence; outcome-based endpoints [35,36,37,38]
**Generalizability and Data**	Single-center data, drift, poor data quality	Heavy reliance on MIMIC III and IV, and eICU v2.0 datasets; temporal drift and noisy ICU data degrade performance	Multicenter and federated learning; continuous auditing and recalibration; standardized ETL pipelines [35,39,40,41]
**Model** **Transparency**	Limited validation, interpretability, reproducibility	Few models externally validated; “black box” DL/RL models; limited code/data sharing	Mandatory external validation; explainable AI; open-science practices [36,37,38,42,43]
**Clinical** **Integration**	Workflow mismatch, automation bias	Poor EHR integration; alert fatigue; over-reliance on AI outputs	Clinician co-design; EHR-embedded tools; human-in-the-loop decision support [38,40,41]
**Ethical and** **Legal**	Accountability, bias, privacy concerns	Unclear liability; demographic bias in training data; data-sharing constraints	Governance frameworks; bias auditing and subgroup reporting; privacy-preserving ML [35,39,41]
**Sustainability**	Poor post-deployment oversight; overly broad tools	Limited monitoring after deployment; broad models underperform	Lifecycle performance monitoring; task-specific AI systems [37,41,44]

**Table 2:** Selected studies on AI-enabled systems to improve sepsis recognition and sepsis bundle compliance.

Author	Setting	AI Methods	Data Inputs	Purpose	Results
**Cooper (2020) [45]**	Community hospital, inpatient-wide screening	Logistic regression (automated screening model)	Six routinely collected sepsis-related variables	Early sepsis identification to facilitate timely bundle initiation	AUROC 0.857; screened 100% of inpatients and delivered alerts without manual nursing intervention (process capability to support bundle initiation)
**Sendak (2020) [10]**	Health-system deployment (ED/inpatient workflows), *Sepsis Watch*	Deep learning (sepsis detection + management platform)	EHR data streams (structured clinical data; model-driven risk)	Improve sepsis detection and support management workflows	Described as a platform used to improve compliance with recommended sepsis treatment guidelines
**Goh (2021) [47]**	Multicenter EHR + clinical notes	Hybrid ML + NLP (SERA)	Structured EHR data + unstructured clinical notes	Early prediction/diagnosis of sepsis (e.g., 12 h prediction)	Developed AI algorithm using structured + notes for sepsis prediction/diagnosis; reported high predictive performance
**Nemati (2018) [50]**	ICU real-time prediction	Interpretable ML model (“AI Sepsis Expert”)	Real-time ICU data	Predict sepsis onset ahead of clinical recognition	Predicts sepsis 4–12 h prior to clinical recognition with AUROC values in the range of 0.83–0.85
**Adams (2022) [55]**	Multi-site deployed sepsis alert (TREWS)	Machine learning early warning system	EHR-derived clinical data used by deployed alert	Earlier recognition and prioritization of sepsis care	Prospective multi-site cohort showing clinician response to an alert within 3 h resulted in reduced in-hospital mortality by 18.7%
**Warstadt (2022) [51]**	Emergency department (quality initiative with EHR tool + education)	EHR-based CDS tool (rules/ordering pathway; not “black box” ML)	EHR tool prompts for bundle elements (lactate, cultures, antibiotics, fluids, reassessment)	Improve ED sepsis identification and management; improve bundle compliance	Tool utilization rose 23.3% → 87.2% and 6 h bundle compliance was 62.2% with tool vs. 37.8% without
**Fixler (2023) [52]**	Multi-hospital EHR deployment	“Predictive learning algorithm” driving CDS tools	EHR-driven risk categories feeding best practice alerts (BPAs)	Increase actionable sepsis CDS engagement and multidisciplinary sepsis management	Higher alert engagement: alert-to-action ratio 16.5% with algorithm vs. 8.4–12.1% for standard BPAs
**Kijpaisalratana (2024) [58]**	Emergency department; cluster-randomized trial	Machine-learning-assisted sepsis alert (MLASA)	Real-time ED clinical/EHR data feeding alert	Enhance timely antibiotics and diagnostic accuracy in ED sepsis	Improved timeliness of antibiotic administration within 1 and 3 h with diagnostic accuracy
**Bhargava (2024) [59]**	5 U.S. institutions; suspected infection (blood culture ordered)	FDA-authorized AI/ML risk score (“Sepsis ImmunoScore”)	Multidomain inputs (demographics, vitals, labs) plus sepsis biomarkers; intended EMR integration	Identify patients at risk of sepsis within 24 h and predict adverse outcomes	Diagnostic AUROC 0.85 derivation, 0.80 internal validation, 0.81 external validation cohort
**Boussina (2024) [56]**	Two EDs (before–after quasi-experimental)	Deep learning (COMPOSER)	EHR-derived features; designed to reduce false alarms	Early sepsis prediction to improve outcomes and care delivery	Deployment associated with 5.0% absolute increased sepsis bundle compliance and 1.9% absolute mortality reduction
**Grooms (2025) [57]**	ED QI project, community hospital	Rule-based “AI” + workflow	Rule logic using ED clinical criteria feeding workflow prompts	Prompt early sepsis management and improve compliance	Implementation resulted in 89.5% compliance to combined antibiotic given, blood culture drawn, and lactate measurement at 3 h. Hospital LOS decreased by 2.3 days and mortality decreased by 22.3%
**Valan (2025) [60]**	External validation across community EDs	*Sepsis Watch* ML model (deep learning)	Static + dynamic EHR data	Validate model performance/clinical utility in new setting	Multisite external validation of *Sepsis Watch* showing AUROC 0.91 to 0.96 for sepsis prediction

**Table 3:** Selected studies of AI for sepsis fluid and hemodynamic management.

Author	Setting	AI Methods	Data Inputs	Purpose	Results
**Celi (2008) [61]**	MIMIC-II (single ICU database); vasopressor pts	Bayesian network	Demographic + physiologic variables from first 24 h	Predict fluid requirement (total fluid on 2nd ICU day)	Accuracy 77.8%
**Komorowski (2018) [62]**	MIMIC-III (train) + eICU (test)	Reinforcement learning policy	Forty-eight variables incl. demographics, vitals, labs, fluids/pressors	Joint fluids + vasopressors over time	Higher estimated policy value than observed clinician policy; lowest mortality when clinician dosing most closely matched AI policy
**Gupta (2021) [63]**	MIMIC-III; 1122 sepsis ICU pts	Human-in-the-loop + inverse classification (classifier + optimization)	EHR covariates used to predict mortality	Personalized intravenous fluid quantity	Estimated ~22% average mortality reduction under model-recommended dosing
**Jeter (2021) [64]**	MIMIC-III; 5366 sepsis pts; hourly data	RL (continuous action) + switching SGLM states	Time-varying clinical variables (hourly)	Fluids + vasopressors for hypotensive episodes (timing/dose)	Agent resuscitated earlier (≈1 h vs. 4 h after diagnosis) with ~3% expected survival improvement
**Su (2022) [65]**	PICMISD; 2705 sepsis pts	RL + Deep Q-learning	27 features (25 state + action fluid balance + outcome)	Direction of fluid therapy and fluid balance over time	Higher learned Q-values associated with lower mortality; identified U-shaped harm at extremes of fluid balance
**Liang (2024) [66]**	MIMIC-III; 412 sepsis patients	RL + neural network	Longitudinal EHR trajectory (vitals/labs + treatment history)	Multi-stage fluid resuscitation dosage	Expected mean SOFA reduction of 23.71% with recommended adequate fluid resuscitation
**Oh (2025) [67]**	MIMIC-IV (dev) + SICdb database (external)	Causal ML	EHR features to estimate individualized treatment effects	Restrictive vs. liberal fluids in sepsis + AKI	Restrictive fluids associated with higher AKI reversal (53.9% vs. 33.2%) and lower 30-day major adverse kidney events (17.1% vs. 34.6%)
**Lin (2019) [68]**	MIMIC-III Sepsis-3; 19,275 pts; 232,929 events	Gradient tree boosting ML	Physiologic parameters around fluid event	Predict urine output response/oliguria after fluids	Oliguria prediction AUROC > 0.86
**Bataille (2020) [69]**	Prospective observational; severe sepsis/septic shock; 100 pts (50 train/50 test)	ML (CART, PLS, NNET, LDA) models on TTE features	Transthoracic echocardiography + physiologic changes	Fluid responsiveness (ΔSV ≥ 15%)	AUROCs: PLR 0.77; CART 0.68; PLS 0.83; NNET 0.83; LDA 0.85
**Kamaleswaran (2021) [70]**	MIMIC-III + matched physiologic data	ML with waveform features (logistic regression)	Clinical data + continuous physiologic waveforms	Predict volume responsiveness in sepsis	With waveform features, AUROC 0.89, compared to AUROC 0.84 for clinical factors without waveform information
**Catling (2023) [71]**	Scoping review of seventy-three studies	Supervised + RL systems	N/A	Cardiovascular resuscitation decision support	RL systems increasingly used for fluids/pressors, but most remain proof-of-concept

**Table 4:** Implementation barriers and mitigation strategies for AI-enabled sepsis care.

Domain	Implementation Barrier(s)	Mitigation Strategy	Practical Sepsis AI Example
**Clinician acceptance, trust, interpretability [10,33,48,77,78]**	Distrust/confusion for ML models vs. rule-based tools; perceived “black box”; low perceived usefulness	Early and continuous clinician engagement; transparent model communication (what it does/does not do); just-in-time education; feedback loops; identify local champions; align tool purpose to clinical priorities	Brief, role-specific training on how to interpret risk scores/alerts + structured feedback mechanism to iteratively refine alert content and thresholds
**Workflow integration and alert burden [10,46,48,77]**	Alert fatigue; wrong recipient; unclear escalation pathway; misfit with existing sepsis workflows and staffing patterns	Co-design the “micro-workflow” around the AI output; define who receives alerts, triage steps, and escalation rules; minimize interruptions; ensure alerts are actionable and time-appropriate	Route alerts to a designated triage staff (e.g., rapid response team or charge nurse) who performs rapid chart review, then escalates to the treating team when indicated
**Data quality, generalizability, and technical readiness [10,33,78]**	Limited/diverse datasets; missingness; drift; poor calibration in local populations; interoperability constraints	Pre-implementation data readiness assessment; local validation (including calibration); “silent” pilot before go-live; ongoing drift monitoring; periodic recalibration; bias checks and mitigation	Run silent predictions for several weeks to compare alert performance vs. clinician recognition, then calibrate the model before activating workflow triggers
**Governance, liability/regulatory, and patient safety [10,33,34,46,78]**	Unclear accountability and liability; privacy/security concerns; risk of unintended harms (e.g., overtreatment, antibiotic overuse)	Establish governance (ownership, oversight, escalation for safety issues); define accountability; document decision support role; audit trails; safety monitoring plan with balancing measures	Monitor both “benefit” metrics (time-to-antibiotics, bundle completion) and balancing metrics (broad-spectrum antibiotic exposure, false-positive escalations)
**Change management, leadership alignment, and stakeholder buy-in [10,16,34,77]**	Misaligned priorities; inadequate leadership support; insufficient change management; resistance to new roles/processes	Executive sponsorship; stakeholder mapping; communication plan; staged rollout; clarify role changes; align with QI goals (i.e., SEP-1 measure compliance)	Implementation steering group (ICU/ED leaders, nursing, informatics, patient safety) sets adoption goals and manages iterative workflow changes
**Workforce capacity, training, and sustainment [10,34,46,48]**	High training burden; staffing limitations and turnover; ongoing maintenance needs; implementation fatigue	Dedicated implementation team; recurring training and onboarding; clear maintenance plan (monitoring cadence, retraining triggers); resource budgeting for sustainment	Monthly model performance and workflow review huddles; refresh training for new clinicians; defined triggers for recalibration

## Data Availability

No new data were created or analyzed in this study. Data sharing is not applicable to this article.

## Abbreviations

The following abbreviations are used in this manuscript:

**Abbreviation**	**Meaning**
AI	Artificial Intelligence
AKI	Acute Kidney Injury
AUROC	Area Under the Receiver Operating Characteristics Curve
BPA	Best Practice Alert
CART	Classification and Regression Tree
CDS	Clinical Decision Support
CDSS	Clinical Decision Support System
CMS	Centers for Medicare & Medicaid Services
COMPOSER	COnformal Multidimensional Prediction Of SEpsis Risk
CONSORT-AI	Consolidated Standards of Reporting Trials–Artificial Intelligence
DL	Deep Learning
ED	Emergency Department
EHR	Electronic Health Record
eICU	Electronic Intensive Care Unit Collaborative Research Database
ETL	Extract, Transform, Load
FDA	Food and Drug Administration
ICD-10	International Classification of Diseases, 10th Revision
ICU	Intensive Care Unit
LDA	Linear Discriminant Analysis
LOS	Length of Stay
MAP	Mean Arterial Pressure
MGP	Multitask Gaussian Processes
MIMIC	Medical Information Mart for Intensive Care database
ML	Machine Learning
MLASA	Machine Learning-Assisted Sepsis Alert
MPC	Model Predictive Control
NLP	Natural Language Processing
NNET	Neural Network
PICMISD	Peking Union Medical College Hospital Intensive Care Medical Information System and Database
PLR	Passive Leg Raise
PLS	Partial Least-Squares Regression
QI	Quality Improvement
qSOFA	Quick Sequential Organ Failure Assessment
RL	Reinforcement Learning
RNN	Recurrent Neural Network
SEP-1	Severe Sepsis and Septic Shock Early Management Bundle
SERA	Sepsis Early Risk Assessment
SGLM	Switching Generalized Linear Model
SIRS	Systemic Inflammatory Response Syndrome
SOFA	Sequential Organ Failure Assessment
SSC	Surviving Sepsis Campaign
TREWS	Targeted Real-Time Early Warning System
TRIPOD-AI	Transparent Reporting of a Multivariable Prediction Model for Individual Prognosis or Diagnosis–Artificial Intelligence
UO	Urine Output
VAE	Variational Autoencoder
